# Induced superconductivity in hybrid Au/YBa_2_Cu_3_O_7−x_ electrodes on vicinal substrates

**DOI:** 10.1038/s41598-025-17434-y

**Published:** 2025-09-02

**Authors:** Irina Gundareva, Jose Martinez-Castro, Frank Stefan Tautz, Gregor Mussler, Abdur Rehman Jalil, Xiao Hou, Detlev Grützmacher, Thomas Schäpers, Matvey Lyatti

**Affiliations:** 1 Peter Grünberg Institute (PGI-9), Forschungszentrum Jülich, 52425 Jülich, Germany; 2 Peter Grünberg Institute (PGI-3), Forschungszentrum Jülich, 52425 Jülich, Germany; 3 Jülich Aachen Research Alliance, Fundamentals of Future Information Technology, 52425 Jülich, Germany; 4 Institute of Experimental Physics II B, RWTH Aachen, 52074 Aachen, Germany; 5 Institute of Experimental Physics IV A, RWTH Aachen, 52074 Aachen, Germany; 6 Peter Grünberg Institute (PGI-10), Forschungszentrum Jülich, 52425 Jülich, Germany; 7 Peter Grünberg Institute (PGI-6), Forschungszentrum Jülich, 52425 Jülich, Germany

**Keywords:** Superconducting properties and materials, Surfaces, interfaces and thin films

## Abstract

Superconducting electrodes are an integral part of hybrid Josephson junctions used in many applications including quantum technologies. We report on the fabrication and characterization of superconducting hybrid Au/YBa_2_Cu_3_O_7−x_ (YBCO) electrodes on vicinal substrates. In these structures, superconducting CuO_2_-planes face the gold film, resulting in a higher value and smaller variation of the induced energy gap compared to conventional Au/YBCO electrodes based on films with the c-axis normal to the substrate surface. Using scanning tunneling microscopy, we observe an energy gap of about 10–17 meV at the surface of the 15- nm-thick gold layer deposited in situ atop the YBCO film. To study the origin of this gap, we fabricate nanoconstrictions from the Au/YBCO heterostructures and measure their electrical transport characteristics. The conductance of the nanoconstrictions shows a series of dips due to multiple Andreev reflections in YBCO and gold, providing clear evidence of the superconducting nature of the gap in gold. We consider the Au/YBCO electrodes to be a versatile platform for hybrid Josephson devices with a high operating temperature.

**Supplementary Information:**

The online version contains supplementary material available at 10.1038/s41598-025-17434-y.

In recent years, several emerging 1D (one-dimensional) and 2D (two-dimensional) materials such as semiconductor nanowires, graphene, or topological insulators have been attracting growing attention. Although these materials possess unique properties, most of them obey the Fermi-Dirac statistics which does not allow for a macroscopic quantum state to appear. However, when they are combined with a superconductor, the macroscopic quantum state can be induced by the proximity effect close to the interface^1^. Having great potential for quantum technologies, such hybrid structures are also exciting objects for fundamental research.

For example, graphene proximitized with a superconductor is a promising platform for studying 2D quantum phase transitions, topological superconductivity, and hybrid devices for quantum computing^2,3^. Furthermore, the interface between a superconductor and a topological insulator is predicted to host Majorana fermions, and many efforts have been made in search of Majorana particle that paves the way to the realization of a topologically protected fault-tolerant quantum computer^4^. The topological state can be realized using an s-wave superconductor coupled to a 1D semiconductor nanowire with a strong spin-orbit coupling and a high g-factor^5–7^. Semiconductor nanowires can also be used as weak links to create Josephson junctions for qubits based on other physical principles such as gatemons or Andreev-level qubits^8^. However, the value of the induced superconducting gap in hybrid devices based on low-*T*_*c*_ superconductors is rather small and typically has µeV scale which makes them vulnerable to external interferences.

It has been theoretically predicted that an alternative approach using high-temperature (high-*T*_*c*_) superconductors with d_x2−y2_-wave symmetry of the order parameter and large anisotropic energy gaps of tens of meV could give far more powerful results^9–13^. The use of high-*T*_*c*_ superconductors can not only increase the operating temperature and stability of hybrid devices but reveals exciting physics. First reports on graphene proximitized with a Pr_2−x_Ce_x_CuO_4_ superconductor have shown signatures of proximity-induced unconventional p-wave pairing in graphene^14^ and are in good agreement with theoretical predictions^15^. Several works are devoted to hybrid structures based on Bi_2_Sr_2_CaCu_2_O_8_ (BSCCO) covered by a thin layer of topological insulators Bi_2_Se_3_ or Bi_2_Te_3_^16–21^. Notably, the investigations of the hybrid structures by angle-resolved photoemission spectroscopy (ARPES) and scanning tunneling microscopy (STM) have given controversial results, and the question of whether one can observe an induced gap in the topological insulators on top of a BSCCO superconductor is still under discussion.

The attempts to proximitize graphene with YBa_2_Cu_3_O_7−x_ (YBCO) superconductor deposited on the conventional substrates showed only hints of induced superconductivity^22,23^. On the one hand, as shown for example for a graphene/YBCO interface^22,23^many emerging materials for the hybrid Josephson junctions have poor compatibility with oxide superconductors. Direct coupling of these proximitized materials to the high-*T*_*c*_ superconductor leads to an inferior interface with low transparency and correspondingly, a large drop of the induced energy gap Δ_ind_ in the interface layer (upper panel in Fig. 1a). A mediating layer between the proximitized material and the oxide superconductor can solve this problem. The mediating layer has to provide a small order parameter attenuation and transparent interfaces to both of the contacting materials, resulting in the larger induced energy gap in the proximitized material (lower panel in Fig. 1a). On the other hand, similar to the other cuprate superconductors, the YBCO has a layered structure with superconducting CuO_2_ planes, as shown in Fig. 1b. The larger the contact area between the CuO_2_ planes and the proximitized layer, the higher the magnitude of the induced superconducting gap is expected. All earlier works on hybrid devices with high-*T*_*c*_ superconducting electrodes are based on the films where the c-axis is normal and the CuO_2_ planes are parallel to the film surface (so-called c-axis oriented films)^24–26^. The sketch of such an electrode is shown in Fig. 1c. The mediating layer in such electrodes is coupled to the CuO-chains where the order parameter is significantly reduced compared to CuO_2_-planes. Therefore, the induced energy gap in these devices is expected to be rather small compared to the intrinsic YBCO energy gaps. The finite surface roughness of the films may provide a small contact area between the CuO_2_ planes and the mediating layer that results in the small and strongly spatially inhomogeneous induced energy gap as experimentally shown for Au/YBCO heterostructures^27^. Such a configuration of the electrodes gives only a hint of induced superconductivity in proximitized material but could hardly ever allow the development of a reproducible hybrid device.

**Fig. 1:**
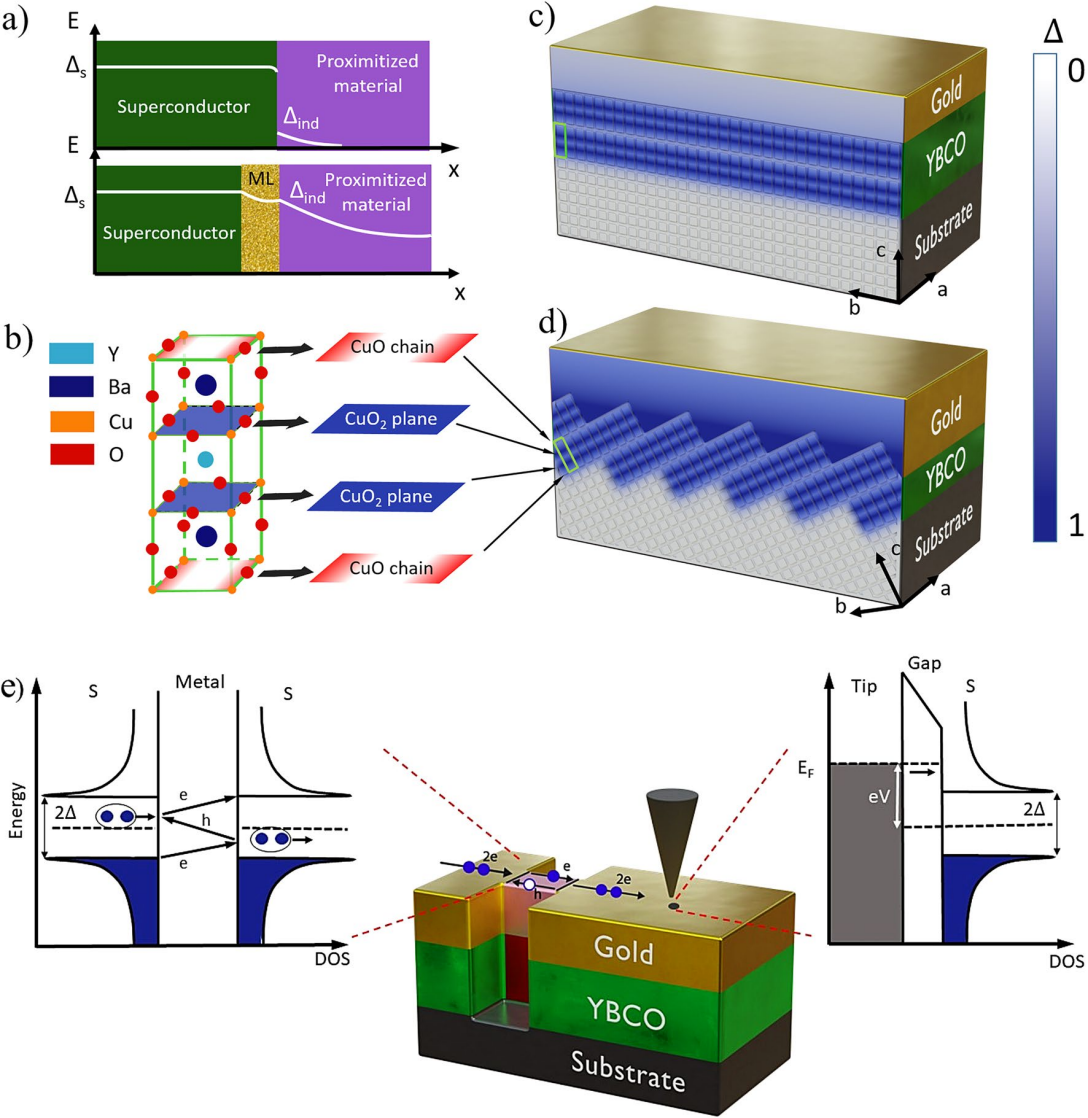
Au/YBCO electrodes. (**a**) Evolution of the order parameter in the hybrid heterostructure, where ML is a mediating layer, Δ_s_ and Δ_ind_ are an energy gap in a superconductor and an induced energy gap, respectively. (**b**) YBCO unit cell. (**c**) Sketch of the conventional hybrid high-*T*_*c*_ electrode, where the c-axis is normal and the CuO_2_ planes are parallel to the film surface. (**d**) Sketch of the hybrid device based on vicinal substrates, where the c-axis is inclined towards the substrate surface normal. A schematic illustration of the order parameter variation in (**c**) and (**d**) is shown in a scale bar on the right side. (**e**) A schematic representation of an STM (right side) and MAR spectroscopy (left side) used to study induced order parameter in gold. Normal-state domains in the constriction neck appearing in the YBCO and gold layers when the bias current exceeds the critical current are indicated in red and pink, respectively. Superconductor/normal metal/superconductor interfaces in the gold layer are indicated by solid black lines. The black arrow in the right panel shows a tunneling current. S indicates the superconductor. 3D images were created in Blender 3.2.14 (www.blender.org).

*Concept*. Here, we propose an alternative approach to the fabrication of the high-*T*_*c*_ superconducting electrodes for hybrid devices based on superconducting films deposited on vicinal substrates. The c-axis of the film grown on the vicinal substrate is inclined towards the substrate surface normal (Fig. 1d). The superconducting CuO_2_ planes have direct access to a film surface, providing better conditions for the injection of Cooper pairs into the material where induced superconductivity is required. Such a configuration is expected to have a larger energy gap and smaller lateral variation of the order parameter at the superconducting film surface. Additionally, no variation in the magnitude of the order parameter is expected across the thickness of the normal metal if its double thickness is less than the corresponding coherence length^28^. In our work, we use gold as the mediating layer because it meets the abovementioned requirements and has a large normal coherence length *ξ*_*n*_ resulting in a reduced spatial variation of the order parameter^29^. It is worth noticing that due to the large mismatch in carrier densities between Au and YBCO, the proximitized gold layer has unpaired quasiparticles even at zero temperature, which limits the use of such an electrode for quantum applications. The sketch of the novel hybrid superconducting Au/YBCO electrode with the gold mediating layer is shown in Fig. 1d.

We perform a comprehensive study of this hybrid superconducting Au/YBCO electrode using STM and multiple Andreev reflection (MAR) spectroscopy, as shown in Fig. 1e. The STM provides information on the density of states at the surface of the studied material with nanometer spatial resolution (right panel in Fig. 1e), while the MARs in the nanoconstriction conductance (left panel in Fig. 1e) are an indicator of the superconducting nature of the observed energy gap.

## Results and discussion

### STM measurements

**Fig. 2:**
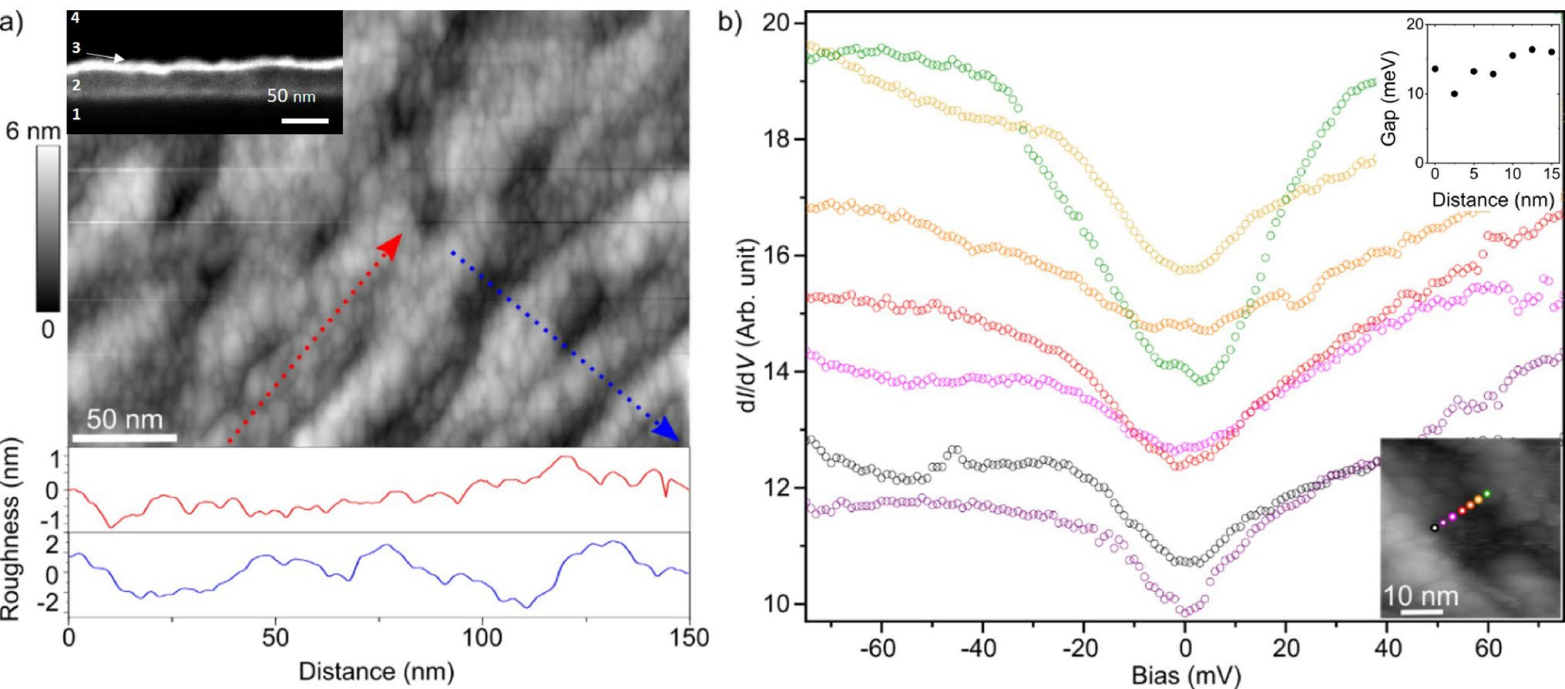
Surface morphology and spectroscopic properties of the Au/YBCO surface. (**a**) Scanning tunneling micrograph of an Au/YBCO heterostructure on a vicinal NGO substrate (upper panel) and surface profiles along and perpendicular to the terraces (red and blue dotted arrows) (*V*_set_ = 1 V, *I*_set_ = 130 pA). The inset shows a scanning electron micrograph of an Au/YBCO heterostructure cross-section. The NGO substrate, YBCO film, Au film, and electron beam deposited Pt film are numbered 1, 2, 3, and 4, respectively. (**b**) Differential conductance spectra taken at different positions of the sample. The spectra are offset vertically for clarity. The inset shows the measurement points (*V*_set_ = 100 mV, *I*_set_ = 2 nA, *V*_mod_ = 1 mV). The colour of each dot corresponds to the colour of the differential conductance spectra taken at this point. The extracted gap values are shown in the upper insert.

We fabricated a number of Au/YBCO heterostructures consisting of 30 to 35-nm-thick epitaxial YBCO films deposited on vicinal SrTiO_3_ (STO) and NdGaO_3_ (NGO) substrates and covered in situ with a 15-nm-thick gold layer. Since a low film roughness is preferable for STM measurements, we selected the heterostructures on NGO substrates for this part of our study, as they have slightly lower roughness than those on STO substrates. Figure 2a shows a representative surface STM topography of the Au/YBCO heterostructure fabricated on a (110) NGO substrate with 10.5° miscut. Along with a granular structure of the gold film with an average grain size of 10 nm, elongated parallel terraces in the direction of the red arrow are observed. These terraces are formed due to the step-flow growth of the YBCO films on the vicinal substrates^30–33^ (see the X-ray diffraction analysis of the film in Supplementary Fig. S1 and the transmission electron microscopy image (TEM) of the film in Supplementary Fig. S2). To avoid a large step-bunching during the step-flow growth and achieve a low roughness of the Au/YBCO electrode surface within a few unit cells, we optimized the substrate surface treatment and sputtering parameters^34,35^. The corresponding root-mean-square roughness of the Au/YBCO electrode surface along and perpendicular to the terraces is 0.29 and 0.84 nm, respectively. A scanning electron micrograph (SEM) of an Au/YBCO electrode cross-section is shown in the inset of Fig. 2a. The analysis of the cross-section confirms the thickness of the gold layer and shows that its surface topology follows that of the YBCO film. We performed differential conductance spectroscopy (d*I*/d*V*) at a temperature *T* = 10 K following a line between two terraces (see Methods for more details on the STM measurements) in a span of 20 nm (inset in Fig. 2b). To avoid oxygen depletion in the YBCO, we did not anneal the sample before measurements. The d*I*/d*V* spectra are V-shaped without distinct coherence peaks. They demonstrate an energy gap varying from 10 to 17 meV at the surface of the gold layer, as shown in the upper insert in Fig. 2b, with an average value of 14.0 ± 2.3 meV (more examples of d*I*/d*V* spectra are available in Supplementary Fig. S3). The gap magnitude at the gold surface 2b does not demonstrate an exponential decrease with increasing distance from the YBCO grain, as reported for the YBCO/Au heterostructures based on YBCO films with the c-axis normal to the substrate surface^27^which is in favour of the proposed vicinal film approach. Moreover, the spectra show no significant correlation of the induced energy gap value with the gold grain size, expected for a situation when the Coulomb blockade is the origin of the observed energy gap^36^. Here, we determine the value of the energy gap as half of the distance between a dip and kink values of the spectra in Fig. 2b following the work of Stepniak et al.^37^where STM measurements using tungsten and niobium tips are compared. The energy gap value may vary at the gold film surface because the normal coherence length in gold, *ξ*_*n*_ ≈ 24 nm at the measurement temperature *T* = 10 K, is comparable with the terrace width. The normal coherence length in the gold layer was estimated as *ξ*_*n*_ = (ℏ*V*_*F*_*l/6*π*k*_*B*_*T*)^1/2^ (Equation 1), where ℏ is the reduced Planck constant, *k*_*B*_ is the Boltzmann constant,, and *V*_*F*_ = 1.4 × 10^6^ m/s is the Fermi velocity in gold taken from the literature^38^. Our estimation is based on assumption that the elastic mean free path *l* ≈ 10 nm in the gold film is governed by grain boundary scattering. We do not observe zero-bias conductance peak which may either indicate an absence of the (110) facets facing the Au/YBCO interface or be consistent with the isotropization of the d-wave symmetry of the order parameter in the diffusive metal^39–41^. Although such isotropization, predicted by Tanaka and Golubov^39,41^, can be very attractive for quantum applications, we cannot conclude from our experimental data about the symmetry of the order parameter in the Au film proximitized by YBCO, since the *dI/dV* spectra of the diffusive metal have the same V-shape both in the case of contact with the s- or d-wave superconductor^37,40,42^.

The anisotropic pairing symmetry in high-*T*_*c*_ cuprate superconductors makes the interpretation of the density of states more complicated in comparison to BCS (Bardeen-Cooper-Schrieffer) superconductors^43^. Sometimes it leads to the question of whether the observed dip in the density of states is due to superconductivity because STM measurements provide information on the single-particle excitation spectrum. For example, recent ARPES and STM studies of the topological insulator Bi_2_Se_3_ grown on BSCCO show discrepancies^16–21^ which have been recently resolved. The apparent energy gap is the consequence of the dynamic Coulomb blockade^44^. However, in our work we exclude Coulomb blockade as the origin of the observed gap since our measurements are performed in a continuous 15-nm-thick gold film, as opposed to metallic nanoislands or disordered atomically thin wetting layers^36,42,45^. To study the origin of the energy gap and get further insight into the physics of the induced superconductivity in the Au/YBCO electrodes, we fabricated Au/YBCO nanoconstrictions and investigate their electrical properties.

### MAR spectroscopy

The Andreev reflection is a phenomenon specific to the charge transfer through the normal metal-superconductor interface. A normal electron with energy below the superconducting gap energy Δ_S_ is retroreflected from this interface as a hole, creating a Cooper pair at the Fermi level of the superconductor and providing an additional charge transfer compared to the electrons with energies above Δ_S_. Within the framework of the Blonder-Tinkham-Klapwijk model^46^a nanoconstriction can be considered as a superconductor-normal metal-superconductor (SNS) junction where the voltage at currents above the critical current is developed across the dissipative neck region. According to the theoretical approach to the SNS junction, each quasiparticle undergoes MAR before it is scattered or leaves the pair potential well, as shown schematically in Fig. 1e. If a quasiparticle undergoes *n* Andreev reflections, then *ne* charges are transferred through a normal metal-superconductor boundary in addition to the initial one, and the SNS junction current is enhanced due to Andreev reflection. Here, *n* is an integer and e is the electron charge. As a consequence of MAR, conductance curves show a series of features at voltages *V* = 2Δ/*ne*. The measurements of the Andreev reflection spectrum give clear evidence of the superconducting nature of the energy gap and provide information on its magnitude^47,48^.

**Fig. 3:**
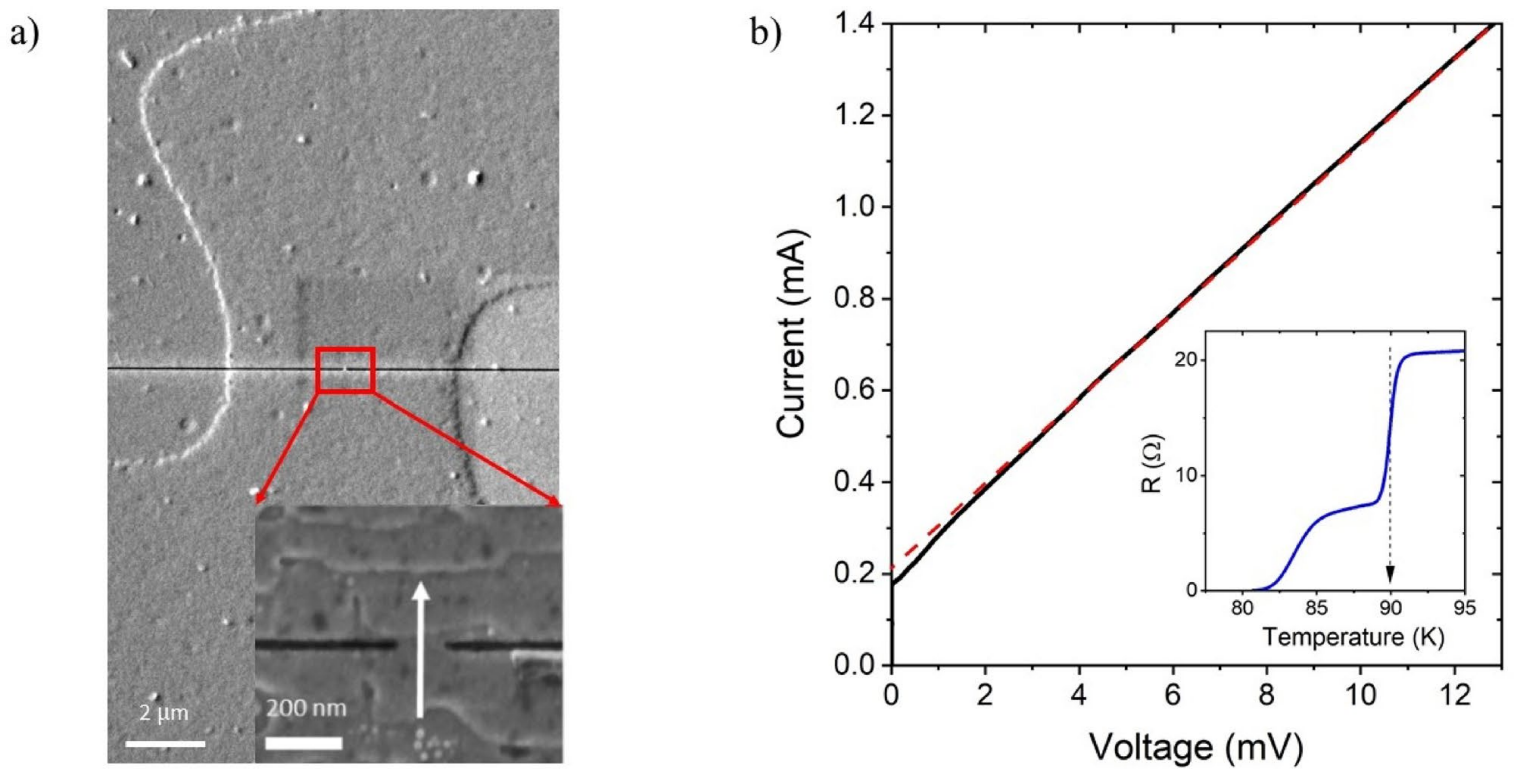
Structural and electrical characteristics of the nanoconstriction. (**a**) SEM image of a microbridge with a nanoconstriction made by FIB. The inset shows the nanoconstriction with a width of 100 nm. The current flows perpendicular to the terraces and is indicated by the white arrow. (**b**) Typical *IV*-curve of a nanoconstriction with a width of 100 nm oriented across the terraces; the extrapolation by dashed red line shows an excess current. The inset shows a typical curve of a transition from normal to superconducting state; the transition temperature of the YBCO electrodes is indicated by the dashed black arrow.

60 to 495-nm-wide and 40 to 60-nm long Au/YBCO nanoconstrictions were fabricated along and perpendicular to the terraces formed by the step-flow growth by focused ion-beam milling or by electron-beam lithography combined with ion-beam etching (see Methods). The constrictions were made as short as possible to reduce the scattering in the constriction neck area. The thickness of the gold layer was chosen to be, on the one hand, smaller than the half of the estimated normal coherence length in Au ξ_n_(4 K) ≈ 37 nm to get uniform magnitude of the order parameter across the gold film thickness and, on the other hand, thick enough to prevent an expansion of the normal-state domain in the nanoconstriction neck at high voltage biases. Nanoconstrictions patterned along the terraces had high critical current densities *J*_*c*_=*I*_*c*_/*Wd* up to 82 MA/cm^2^, confirming that the YBCO film does not degrade after the nanoconstriction patterning. Here, *W* is the width of a nanoconstriction and *d* is the thickness of a YBCO film. No difference in the critical current density was observed for the nanoconstrictions fabricated on NGO and STO substrates. In this work, we study the electrical transport of the nanoconstrictions patterned across the terraces, which have several times lower critical current densities compared to those patterned along the terraces. Such an orientation of the nanoconstriction is beneficial for reducing the heating effects and, consequently, the size of the normal-state region, which, in the framework of the hotspot model^49^depends on the square of the critical current density. The smaller size of the normal-state region provides less scattering, and, correspondingly, more favorable conditions for observing the MAR. Figure 3a and the inset of Fig. 3a show a representative SEM image of a microbridge with a nanoconstriction and its zoomed image, respectively.

A representative resistance temperature dependence *R*(*T*) (the inset in Fig. 3b) has a two-step transition as a result of the weak-link behaviour of a nanoconstriction. The first resistance drop with the onset at 90.5 K occurs due to the superconducting transition of the large-size electrodes of the nanoconstrictions. The constriction itself switches to the zero-resistance state at the lower temperature around *T* = 81 K. Since the decrease in the zero-resistance temperature was not accompanied by a decrease in the critical current density at low temperatures, we attribute it to the thermally activated phase slippage rather than damage to YBCO film during nanoconstriction fabrication.

A representative current-voltage (*IV*) curve of a 100-nm-wide nanoconstriction is shown in Fig. 3b. The nanoconstriction has a normal-state resistance *R*_*n*_ = 14.5 Ω at the voltage *V* ≥ 40 mV and a critical current *I*_*c*_=176 µA at a temperature of 4.2 K. Having a linear dependence above the critical current, the *IV* curve demonstrates neither voltage steps characteristic of phase slippage^50–52^nor power law dependence *V* ~ *I*^*α*^ due to flux flow^50^. Thus, we conclude that the appearance of the normal-state region in the nanoconstriction neck is a dominant mechanism of the resistive state at currents above the critical current. The size of this region is stabilized due to the high thermal conductivity of the gold film. The value of the normal-state resistance at the high voltage bias is slightly higher than the phase-slip resistance at temperature close to *T*_*c*_ that indicates that the entire nanoconstriction is in the normal state and the length of the normal state domain is close to the nanoconstriction length. Therefore, any crystal defects within the nanoconstriction area do not affect its conductance nonlinearity at high current biases. Our constrictions can be considered as an SNS junction at a current above the critical current.

**Fig. 4:**
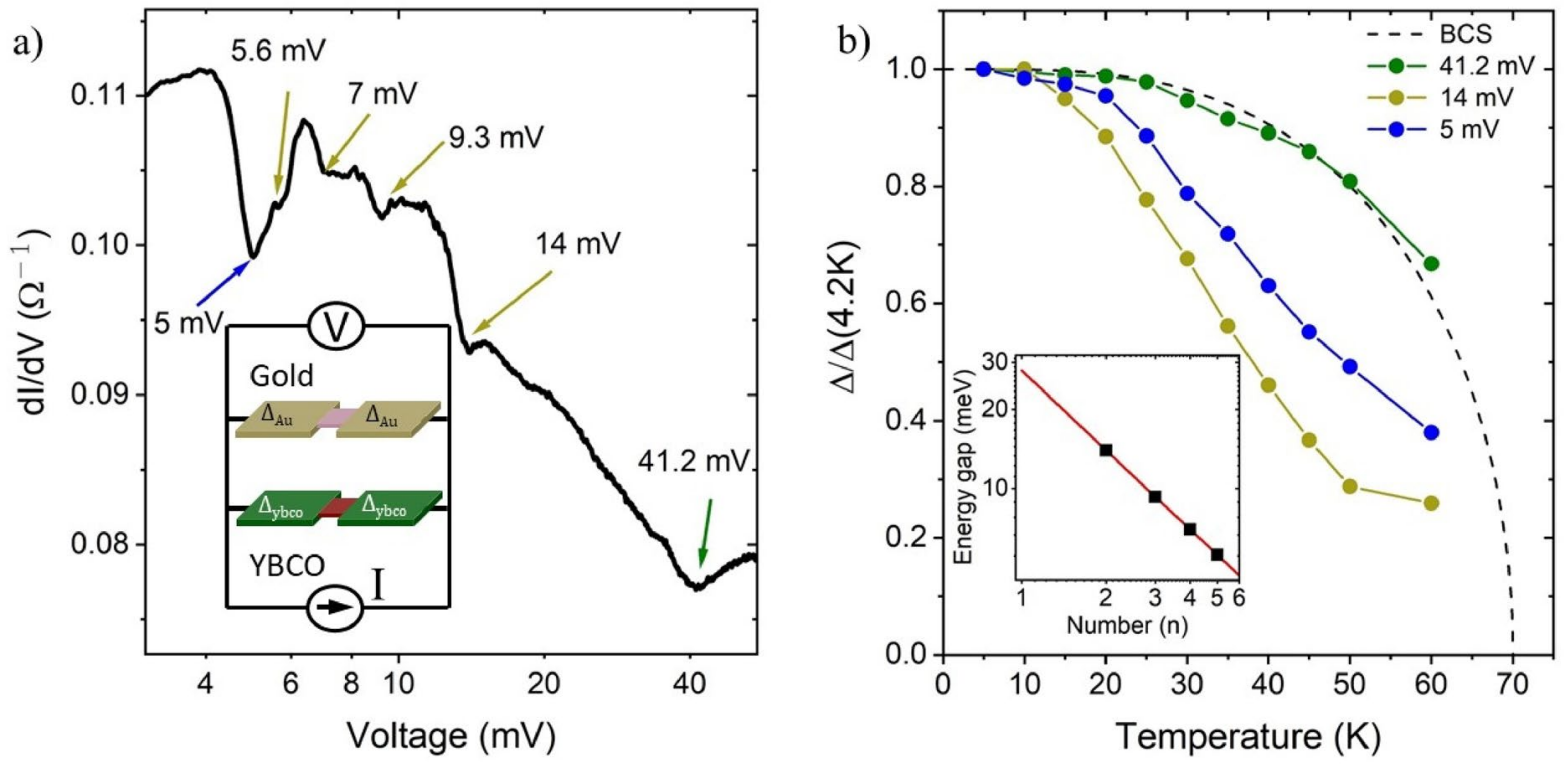
Andreev spectroscopy of the superconducting energy gaps in an Au/YBCO heterostructure. (**a**) Conductance of a 100-nm-wide Au/YBCO nanoconstriction at *T* = 4.2 K. In the inset, an Au/YBCO constriction is presented as a parallel connection of two SNS junctions. A normal state area of the constrictions at the current above the critical current for YBCO and gold is shown in red and pink, respectively. (**b**) Temperature dependence of the normalized energy gaps in Au/YBCO heterostructure. The BCS model prediction is shown by the dashed black line. The inset shows the positions of the conductance dips indicated by dark-yellow arrows. The solid red line represents the *V*_*n*_ *= 2Δ*_*1*_*/ne* dependence with Δ_1_ = 14 meV.

The normal-state YBCO and gold regions are highlighted in red and pink, respectively, in Fig. 1e and the inset of Fig. 4a.

For MAR spectroscopy we chose bridges with the width ranging from 60 to 170 nm. A representative conductance curve of the 100-nm-wide nanoconstriction measured at *T* = 4.2 K shows a nonmonotonic step-like behavior with a number of conductance steps with corresponding dips (Fig. 4a). Here, we follow the numerical simulations of Popovich et al.^53^ for the SNS junctions with transparent normal metal-superconductor interfaces to assign the position of the conductance dips with the nth MAR harmonic at *V* = 2Δ/*ne.* The position of a smeared dip at *V* = 41.2 mV indicated by the green arrow is close to the expected values of Δ/e corresponding to the energy gap in the b-axis direction of YBCO Δ_b_ = 44 meV^54^. The vicinal YBCO films used in this work are twinned and the normal metal-superconductor interface which appears in the nanoconstrictions at currents above the critical current is rounded^55^. Therefore, despite this nanoconstriction demonstrates one conductance dip due to the intrinsic energy gap in YBCO, one can observe the conductance dips associated with the energy gaps either in one antinodal direction Δ_a_ or Δ_b_ or both of them (see Supplementary Fig. S4). The temperature dependence of the Δ_b_ energy gap, shown by the dashed black line in Fig. 4b, is close to the BCS theory prediction and is consistent with the BCS-type of Δ(*T*) dependence reported for the YBCO Josephson junctions^56^. In addition, Fig. 4a shows a series of low-voltage conductance dips indicated by the dark-yellow arrows. Their positions are in perfect agreement with the *V*_*n*_ = 2Δ_1_/*ne* dependence with Δ_1_ = 14 meV shown by the red line in the insert in Fig. 4b for integer *n* from 2 to 5. The temperature dependence of this gap is different from that of the intrinsic YBCO energy gap (Fig. 4b). We assign the energy gap with Δ_1_(4.2 K) = 14 meV to the induced superconducting gap in the gold layer. The sharpness of the conductivity dips associated with the induced order parameter suggests a small variation in its magnitude in gold, otherwise, the dips would be smeared. The magnitude of this gap ranging from 10.5 to 15.3 meV for different nanoconstrictions is close to that measured with STM at the gold layer surface. The ratio between the induced and the corresponding intrinsic superconducting energy gap ranges from 0.30 to 0.41 (see Fig. S4 in Supplementary Materials).

Moreover, we observe a pronounced dip at *V* = 5 mV with temperature dependence different from the abovementioned dips with energy gaps of Δ_b_(4.2 K) = 41.2 meV and Δ_1_(4.2 K) = 14 meV. This dip at voltages ranging from 2.5 to 5 meV is systematically observed for many of our samples. To exclude the second weak link, which may appear in the constriction area, as an origin of this dip, we compare the temperature dependences of the critical current *I*_*c*_ and this dip and find that they are different (see Supplementary Fig. S5). Therefore, we assign this conductance dip to the Δ_2_ energy gap. On the one hand, it was theoretically predicted that so-called surface states appear at the atom layer close to the surface of a metal film^57^. Later, Wei et al.^58^. experimentally showed that a superconductor can induce bulk and surface energy gaps in gold. Then the dip with Δ_2_(4.2 K) = 5 meV may correspond to the induced energy gap in the surface states of gold. On the other hand, the amplitudes of the conductance steps at *V* = 5 mV and *V* = 14 mV are close. Therefore, the Δ_2_ energy gap may belong to the bulk gold and can be induced by the YBCO energy gap in the *c*-axis direction^59,60^. To clarify the nature of this gap, further investigations are required.

Having obtained the value of the induced energy gap with two independent techniques, we would like to estimate the induced superconducting gap theoretically. Sharoni et al.^27^ studied the proximity effect in gold films atop the c-axis oriented YBCO films and found that the induced superconducting energy gap exponentially decays over the thickness of the gold film as $\:{{\Delta\:}}_{Au}\left(d\right)={{\Delta\:}}_{0}{e}^{-d/{\xi\:}_{n}}$, where Δ_0_ = 15 meV is the superconducting energy gap at the Au/YBCO interface, *ξ*_*n*_ is the normal coherence length in metal, and *d* is the distance from the a-axis YBCO surface.

If such an exponential decay was in our system, we would observe smeared MAR conductance steps. However, it contradicts our experimental data.

To obtain a rough check of our results as compared to the other theoretical considerations, we resort to the McMillan model of tunneling between a superconductor and a normal metal where the homogeneous induced order parameter in the normal metal is assumed^28^. In this model, the energy gap Δ_n_ induced in the normal metal film of thickness *D*_*n*_ by the superconducting film of thickness *D*_*s*_ and the energy gap Δ_s_ can be estimated as Δ_n_ = Δ_s_/[1 + *Γ*_*s*_*/Γ*_*n*_] (Equation 2), where *Γ*_*n*_ and *Γ*_*s*_ are the scattering rates in the normal and superconducting films, respectively. First of all, McMillan’s model assumes a tunneling barrier at the normal metal-superconductor interface. The large mismatch in the Fermi velocities of gold *v*_*F*_*(*Au*) =* 1.4·10^6^ m/s^38^ and YBCO *v*_*F*_*(*YBCO*) =* 2·10^5^ m/s^61^ favours the applicability of McMillan´s model in terms of tunneling. Secondly, this model does not include the disruption of Cooper pairs in the normal metal at non-zero temperature that limits its applicability to our system to the lowest temperatures, at which the normal-state coherence length in Au is larger than the doubled film thickness. Within the simplified ballistic approach, the scattering rate in the gold film can be roughly estimated as *Γ*_*Au*_ ≈ ℏ*v*_*F*_(Au)*/2D*_*Au*_, where ℏ is the reduced Plank’s constant and *D*_*Au*_ = 15 nm is the gold film thickness. Such an approach can be used in our case since gold atop YBCO has a polycrystalline structure (see the TEM image of the film in Supplementary Fig. S2). From the available experimental data for the cuprate superconductors BSCCO and LSCO^62,63^we find the scattering rates in the surface layer of these superconductors to be roughly equal to ℏ*v*_*F*_*/ξ*, where *ξ* is the in-plane coherence length. Then, due to the large scattering rate, once the quasiparticle crossed the Au/YBCO interface from Au into YBCO, it has a high probability of being reflected back to Au while scattering in the superconducting layer with a thickness of the order of *ξ*. Therefore, we roughly estimate the scattering rate in YBCO as *Γ*_*YBCO*_ ≈ℏ*v*_*F*_(YBCO)*/2ξ*_*ab*_, where *ξ*_*ab*_ = 1.3 nm is the in-plane coherence length in YBCO^64^. Substituting the abovementioned values of *Γ*_*Au*_ and *Γ*_*YBCO*_ in Eq. 2, we obtain Δ_Au_(4 K) ≈ Δ_YBCO_/[1+(*D*_*Au*_*v*_*F*_(YBCO)/*ξ*_*ab*_ *v*_*F*_(Au))] ≈ 11–17 meV (Eq. 2) which is in good agreement with our experimental results. Here, Δ_YBCO_ = 29–44 meV is the superconducting energy gap in YBCO^54^. Not all of the assumptions of the McMillan model are completely fulfilled in our case. The Macmillan model assumes that the doubled thickness of both the superconductor and the normal metal is smaller than the corresponding coherence lengths. However, this is not valid for YBCO at all temperatures. Therefore, the McMillan model cannot predict the magnitude of the superconducting gap in YBCO. Also, the Macmillan model does not include the surface roughness, which may lead to the small variation in the magnitude of the order parameters between different gold grains. Nevertheless, it can serve as a reasonable estimate of the energy scales in the gold film if it is thin enough. To further demonstrate the consistency of the McMillan model model for Au/YBCO heterostructures, we fit the values of the induced energy gap measured by Sharoni et al.^27^. at the surface of the gold film above the a-plane facets of YBCO with Eq. 2. Here we use Δ_YBCO_ and *ξ*_*ab*_ as free parameters and obtain Δ_YBCO_ = 25 ± 4 meV and *ξ*_*ab*_ = 1.1 ± 0.3 nm, which are close to the values of the energy gap in the a-axis direction and the in-plane coherence length of YBCO, respectively.

Due to the large scattering rate in YBCO close to the Au/YBCO interface, the layers with the intrinsic and induced energy gaps are spatially separated. Therefore, we can model the Au/YBCO constrictions as a parallel connection of two SNS junctions where the voltage at currents above the critical current is developed across the dissipative neck region connecting both YBCO and gold electrodes, as shown in the inset of Fig. 4a. It allows us, in contrast to conventional SNS junctions, to observe both intrinsic and induced energy scales. The higher number of Andreev reflections observed for the gold part of the nanoconstrictions can be explained by weaker scattering in gold compared to YBCO. Therefore, in this paper, we propose a novel technique to study induced superconductivity using Andreev reflection spectroscopy of multilayer nanoconstrictions. Such a technique may be helpful for objects such as hybrid structures capped by an insulating layer or topological insulators with a bottom superconducting electrode that are difficult to access with conventional surface-sensitive techniques.

The fabrication of hybrid devices sometimes requires elevated temperatures which can lead to YBCO degradation. For example, the fabrication of a YBCO/Au/graphene hybrid device using the standard stamp technique includes the heating of the device up to 180 °C to remove the stamp^65^. To prove the ability of the Au/YBCO electrode to withstand this temperature, we anneal the Au/YBCO electrodes for 10 min at a temperature of 180 °C in oxygen at a pressure of 0.8 bar and find that the change in the critical temperature of the electrodes is 1 K only.

A further increase in the induced energy gap may be possible if substrates with higher vicinality are employed. However, since the oxygen out-diffusion at temperatures below 250 °C occurs mainly along the CuO_2_ planes^66^the higher the inclination angle of the c-axis, the faster the YBCO film loses the oxygen, especially at elevated temperatures. This makes the use of YBCO films with a higher inclination angle of the c-axis challenging for the fabrication of hybrid devices.

In summary, we have fabricated and investigated Au/YBCO electrodes on vicinal substrates. STM measurements show an energy gap ranging from 10 to 17 meV at the surface of the 15-nm-thick gold layer. To study the origin of the energy gap in the gold layer, we fabricated the nanoconstrictions from the Au/YBCO heterostructures and performed Andreev reflection spectroscopy, which confirmed the STM results. To the best of our knowledge, the obtained values of the induced energy gap at the surface of the hybrid Au/YBCO heterostructures are the largest ever observed. It confirms the promise of the new approach to the fabrication of hybrid devices, as a combination of such high-*T*_*c*_ superconducting electrodes with emerging 1D and 2D materials. Low roughness, large induced energy gap, high operating temperature, ability to withstand high magnetic fields, and good compatibility with different proximitized materials make the Au/YBCO electrodes a versatile platform for the development and investigation of hybrid devices in a broad temperature range. We consider Andreev reflection spectroscopy with hybrid nanoconstrictions to be a promising technique to determine the induced energy gap in emerging 1D and 2D materials, including topological insulators.

## Methods

### Heterostructure fabrication

The epitaxial YBCO films were deposited by dc sputtering at a high oxygen pressure of 3.4 mbar on vicinal SrTiO_3_ or NdGaO_3_ substrates with a c-axis inclined by 8 or 10.5 degrees towards the substrate surface normal. Before the sputtering, the substrates were etched with buffered oxide etch for TiO_2_ or GaO_2_ surface termination, respectively. The temperature of the heater was 950 °C during the YBCO film deposition. A deposition rate of 1 nm/minute was calculated from profilometer measurements. After the deposition of a 30–35 nm film, the heater temperature was lowered to 550 °C and the film was annealed for 25 min in pure oxygen at the pressure of 800 mbar. Then the heater temperature was ramped down to room temperature. In the next step, the substrate with the YBCO film was transferred into another sputtering chamber and a 15 nm-thick gold film was deposited in situ by dc magnetron sputtering of the gold target in argon at the pressure of 5·10^−3^ mbar. The temperature of the heater was 90 °C during the gold film deposition. 100-nm-thick gold contact pads were deposited *ex situ* by dc magnetron sputtering at room temperature.

### Nanoconstriction fabrication

The nanoconstrictions were fabricated with a two-step process. In the first step, the Au/YBCO structure was patterned into 13 microbridges with a width of 6 μm by the UV- lithography technique followed by ion beam etching of the gold layer in argon and then chemical etching of the YBCO layer in Br-ethanol solution. The microbridges were oriented along and perpendicular to the terraces formed by the step-flow growth. In the second step, 40 to 60-nm-long nanoconstrictions with widths ranging from 100 to 495 nm were fabricated using focused ion beam milling. To protect a sample from Ga ions while milling, it was covered by a 30-nm-thick layer of PMMA resist, and then a 90 nm-thick layer of gold deposited by dc magnetron sputtering. The protection layer was removed in acetone after the nanopatterning. The details of FIB milling are available elsewhere^52^. The nanoconstrictions with a width below 100 nm were made using an inverse process. In the first step, the nanostructures were fabricated by the electron-beam lithography using the ion-beam etching in argon through the 80-nm-thick CSAR62 resist mask. In the second step, the microbridges were defined by UV-lithography and wet chemical etching using the alignment markers fabricated during the first step.

### Experimental setup

 The electrical characteristics of the current-biased nanoconstrictions were measured by a four-probe technique inside a Dewar insert in a 4–80 K temperature range. The differential resistance of the nanoconstrictions was measured with a lock-in amplifier at a modulation frequency of 10 kHz. The temperature of the sample was maintained by a resistive heater controlled by a Lakeshore 335 temperature controller.

### STM measurements

Scanning tunneling data were acquired in a commercial Createc system in an ultra-high vacuum at a base pressure of *P* < 10^−10^ mbar and a base temperature of 10 K using a tungsten tip. Tunneling spectra were acquired using standard lock-in techniques at *f* = 767 Hz and *V*_mod_ = 1mV. The tungsten tip was chemically etched and flashed at high temperatures to remove any impurities. The Au/YBCO sample was transported in ambient conditions and introduced in UHV after pumping the load lock chamber overnight. No annealing in UHV was performed to avoid thermal degradation of YBCO.

## Supplementary Information

Below is the link to the electronic supplementary material.

Supplementary Material 1

## Data Availability

Data supporting the findings of this manuscript are available from the corresponding author upon reasonable request.
