# Supplementary material for: Induced superconductivity in hybrid Au/YBa2Cu3O7−x electrodes on vicinal substrates
Source: Sci Rep. 2025 Sep 2;15:32282. doi: 10.1038/s41598-025-17434-y (PMC12402209; doi:10.1038/s41598-025-17434-y)
Supplement: Supplementary file 1 — Supplementary Material 1 [file 41598_2025_17434_MOESM1_ESM.pdf]

Supplementary Materials

Induced Superconductivity in Hybrid Au/$YBa_2Cu_3O_{7-x}$ Electrodes on Vicinal Substrates

Irina I. Gundareva[1,3*], Jose Martinez-Castro[2,3,4], F. Stefan Tautz[2,3,5], Gregor Mussler[1,3], Abdur Rehman Jalil[6], Hou Xiao[7], Detlev Grützmacher[1,3], Thomas Schäpers[1,3], Matvey Lyatti[1,3]

[1] Peter Grünberg Institute (PGI-9), Forschungszentrum Jülich, 52425 Jülich, Germany

[2] Peter Grünberg Institute (PGI-3), Forschungszentrum Jülich, 52425 Jülich, Germany

[3] Jülich Aachen Research Alliance, Fundamentals of Future Information Technology, 52425 Jülich, Germany

[4]Institute of Experimental Physics II B, RWTH Aachen, 52074 Aachen, Germany

[5]Institute of Experimental Physics IV A, RWTH Aachen, 52074 Aachen, Germany

[6] Peter Grünberg Institute (PGI-10), Forschungszentrum Jülich, 52425 Jülich, Germany

[7] Peter Grünberg Institute (PGI-6), Forschungszentrum Jülich, 52425 Jülich, Germany

XRD analysis

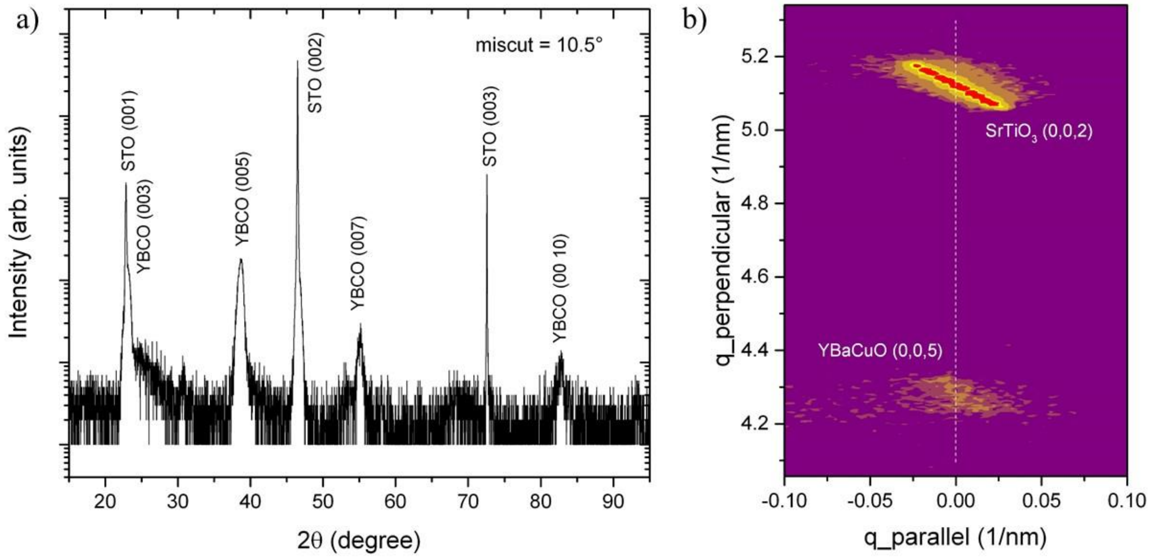

Figure S1. a) XRD 2θ/θ scan of the $YBa_2Cu_3O_{7-x}$ (YBCO)/$SrTiO_3$ (STO) sample. For the measurement, the θ value had to be shifted by 10.5° to accommodate the substrate's miscut. Besides the peaks stemming from the STO substrate, several peaks originating from the YBCO epilayer are seen. These peaks evidence the single-crystal nature of the YBCO film with the (0,0,1) orientation in the growth direction. From the diffraction angles of the (00X) peaks, we calculate the length of the YBCO c-axis parameter as 11.67 Å b) Symmetric reciprocal space map of the STO (0,0,2) substrate peak and the YBCO (0,0,5) epilayer peak. Both peaks are located at $q_{||} = 0$ nm^{-1}, proving that the YBCO epilayer grows parallel on the STO substrate, despite the substantial substrate's miscut.

TEM analysis

Figure S2a demonstrates a representative TEM image of the Au/YBCO heterostructure fabricated on a (110) NGO substrate with an 11.3° miscut. Figure S2b shows a zoomed-in view of the Au/YBCO interface, where lattice fringes in the gold layer reveal the polycrystalline nature of the deposited gold. The thickness of the gold grains is equal to the film thickness. An enlarged view of the YBCO/NGO interface in Figure S2c confirms the epitaxial growth of the YBCO film.

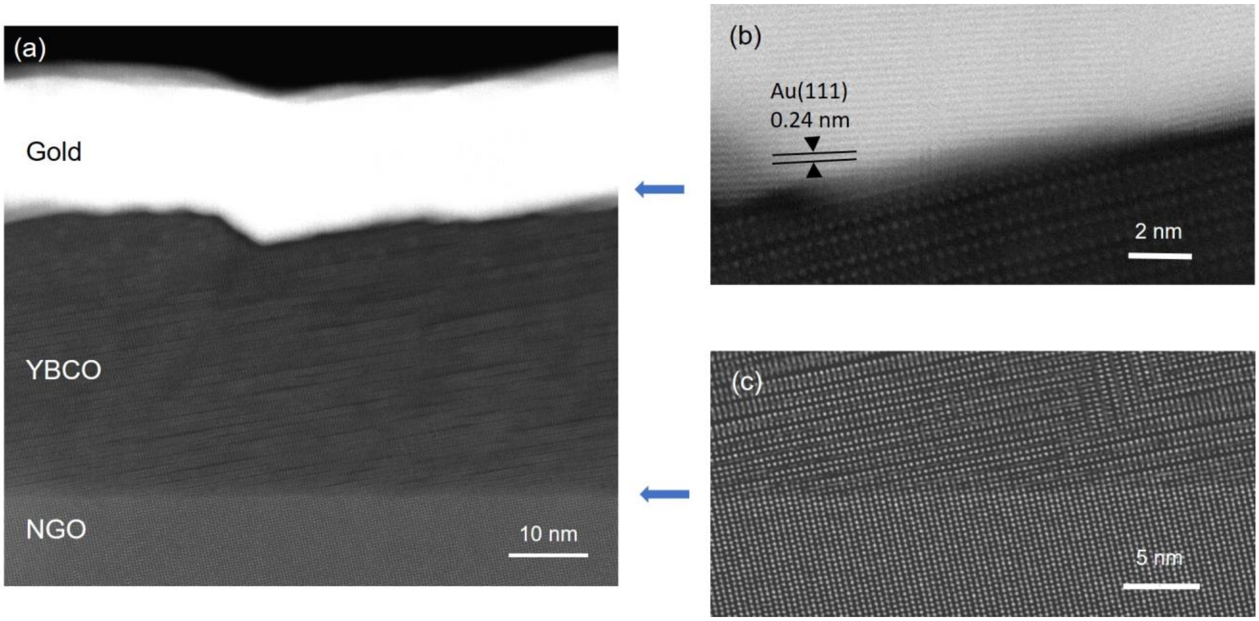

Figure S2. HAADF-STEM images of Au/YBCO heterostructure on the vicinal $NdGaO_3$ (NGO) substrate (a) Overview of the heterostructure: polycrystalline Au, YBCO, and NGO from top to bottom. (b) A zoomed-in view of the Au/YBCO interface. Black lines indicate the Au(111) lattice fringes. The image was blacked out to highlight the gold structure. (c) A zoomed-in view of the YBCO/NGO interface.

STM measurements

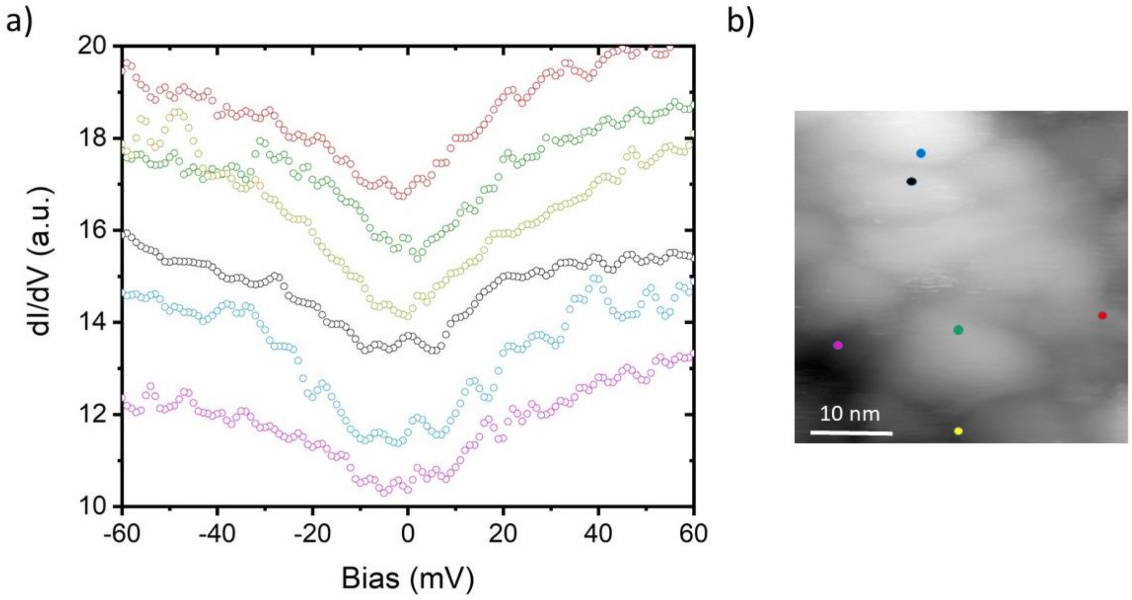

Figure S3. STM measurements. (a) Differential conductance spectra were taken at different positions of the Au/YBCO sample. The spectra are offset vertically for clarity. (b) The map of the measurement points (V_{set} = 80 mV, I_{set} = 2 nA, V_{mod} = 1 mV). The colour of each dot corresponds to the colour of differential conductance spectra taken at this point.

Multiple Andreev reflections in Au/YBCO nanoconstrictions

YBCO possesses d-wave symmetry of the order parameter with different superconducting energy gaps in the **a**-axis and **b**-axis directions, Δ_a =29 meV and Δ_b =44 meV, respectively.[1] One can observe conductance steps corresponding to either the superconducting energy gap Δ_a and Δ_b or both of them due to the twinning of the YBCO films and rounded NS surface in the nanoconstriction neck, which appears at currents above the critical current. We assume that the energy gaps in gold Δ_a^{Au} = 11.5 meV and Δ_b^{Au} = 15.3 meV identified in Figure S4a are induced by the corresponding energy gaps in YBCO Δ_a = 28 meV and Δ_b = 42 meV.

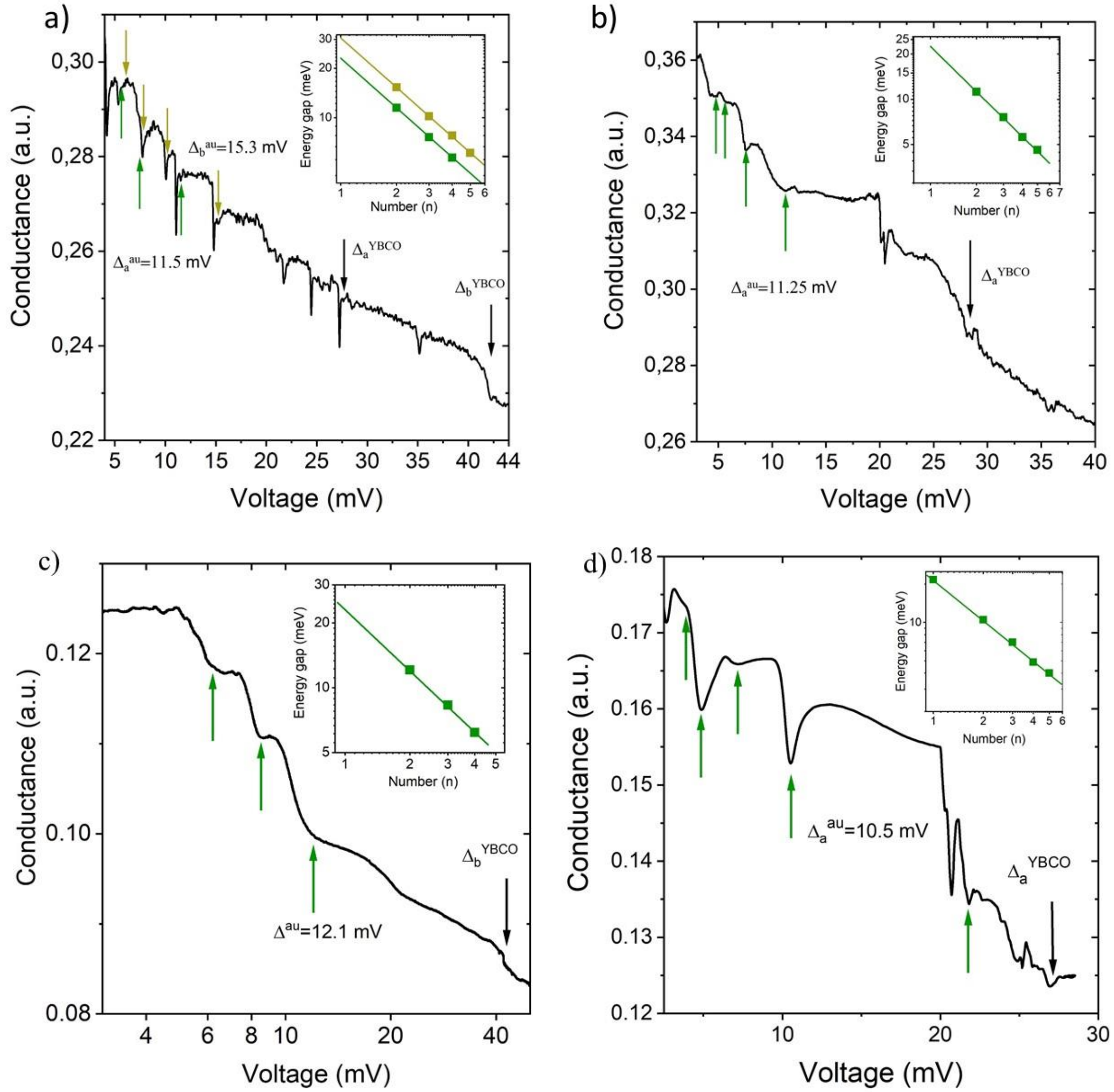

Figure S4. Andreev spectroscopy of the superconducting energy gaps in Au/YBCO heterostructures at the temperature of T = 4.2 K. (a) Conductance of a 90-nm-wide Au/YBCO/STO. Inset shows the positions of the conductance dips indicated by green and dark-yellow arrows, respectively. The solid green and dark-yellow lines represent $V_n = 2\Delta/ne$ dependence with Δ_a^{Au} = 11.5 meV and Δ_b^{Au} = 15.3 meV, respectively. $\Delta_a^{Au}/\Delta_a^{YBCO}$ = 0.41, $\Delta_b^{Au}/\ \Delta_b^{YBCO}$ = 0.36 (b) Conductance of a 60-nm-wide Au/YBCO/STO nanoconstriction. Inset shows the positions of the conductance dips indicated by the green arrows. The solid green line represents $V_n = 2\Delta/ne$ dependence with Δ_a^{Au} = 11.25 meV. $\Delta_a^{Au}/\ \Delta_a^{YBCO}$ = 0.40 (c) Conductance of a 105-nm-wide Au/YBCO/NGO nanoconstriction. Inset shows the positions of the conductance dips indicated by the green arrows. The solid green line represents $V_n = 2\Delta/ne$ dependence with Δ^{Au} = 12.1 meV. $\Delta^{Au}/\ \Delta b^{YBCO}$ = 0.30 (d) Conductance of a 170-nm-wide Au/YBCO/NGO nanoconstriction. Inset shows the positions of the conductance dips indicated by the green arrows. The solid green line represents $V_n = 2\Delta/ne$ dependence with Δ_a^{Au} = 10.5 meV. $\Delta_a^{Au}/\ \Delta_a^{YBCO}$ = 0.39.

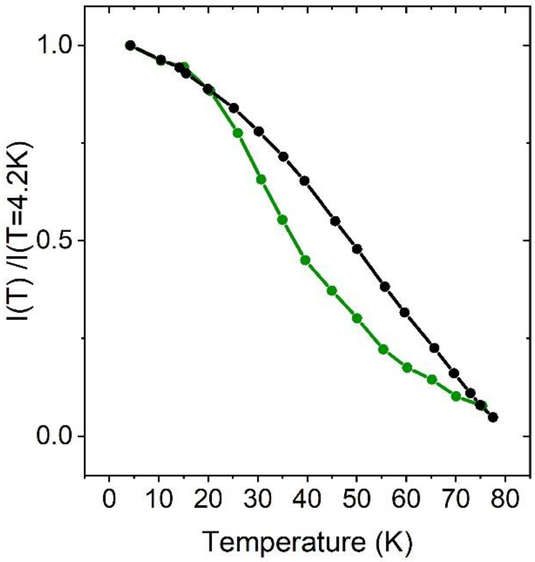

Figure S5. A normalized temperature dependence of the critical current and current position of the low-voltage conductance step associated with Δ_a^{Au} (4.2K) = 10.5 meV for Au/YBCO/NGO nanoconstriction which belongs to the sample in Figure S4(d) are shown in black and green, respectively.

References

1. Lu, D.H. et al. Superconducting gap and strong in-plane anisotropy in untwinned $YBa_2Cu_3O_{7-d}$. *Phys Rev Lett* **86**, 4370-4373 (2001).
